# Risk of Bleeding after Colorectal Endoscopic Resection in Patients with Continued Warfarin Use Compared to Heparin Replacement: A Propensity Score Matching Analysis

**DOI:** 10.1155/2021/9415387

**Published:** 2021-12-17

**Authors:** Katsuaki Inagaki, Ken Yamashita, Shiro Oka, Fumiaki Tanino, Noriko Yamamoto, Yuki Kamigaichi, Hirosato Tamari, Yasutsugu Shimohara, Tomoyuki Nishimura, Yuki Okamoto, Hidenori Tanaka, Takahiro Kotachi, Ryo Yuge, Yuji Urabe, Yasuhiko Kitadai, Kenichi Yoshimura, Shinji Tanaka

**Affiliations:** ^1^Department of Gastroenterology and Metabolism, Hiroshima University Hospital, Hiroshima, Japan; ^2^Department of Endoscopy, Hiroshima University Hospital, Hiroshima, Japan; ^3^Division of Regeneration and Medicine Center for Translational and Clinical Research, Hiroshima University Hospital, Hiroshima, Japan; ^4^Faculty of Human Culture and Science, Department of Health Sciences, Prefectural University of Hiroshima, Hiroshima, Japan; ^5^Medical Center for Translational and Clinical Research, Hiroshima University Hospital, Hiroshima, Japan

## Abstract

The Japan Gastroenterological Endoscopy Society (JGES) guidelines recommend continued warfarin treatment during gastroenterological endoscopic procedures with a high risk of bleeding as an alternative to heparin replacement in patients on warfarin therapy. However, there is insufficient evidence to support the use of warfarin in colorectal endoscopic resection (ER). The present study is aimed at verifying the risk of bleeding after ER for colorectal neoplasia (CRN) in patients with continued warfarin use. This was a single-center retrospective cohort study using clinical records. We assessed 126 consecutive patients with 159 CRNs who underwent ER (endoscopic mucosal resection, 146 cases; endoscopic submucosal dissection, 13 cases) at Hiroshima University Hospital between January 2014 and December 2019. Patients were divided into two groups: the heparin replacement group (79 patients with 79 CRNs) and the continued warfarin group (47 patients with 80 CRNs). One-to-one propensity score matching was performed to compare the bleeding rate after ER between the groups. The rate of bleeding after ER was significantly higher in the heparin replacement group than in the continued warfarin group for both before (10.1% vs. 1.3%, respectively; *P* = 0.0178) and after (11.9% vs. 0%, respectively; *P* = 0.0211) propensity score matching. None of the patients experienced thromboembolic events during the perioperative period. The risk of bleeding after colorectal ER was significantly lower in patients with continued warfarin use than in those with heparin replacement. Our data supports the recommendations of the latest JGES guidelines for patients receiving warfarin therapy.

## 1. Introduction

Colorectal cancer is the third most common cancer in men and the second most common cancer in women worldwide [1]. Globally, the population of older individuals and the number of patients receiving antithrombotic agents (antiplatelet agents and anticoagulants) have increased [2]. Endoscopists are increasingly performing more endoscopic resection (ER) for colorectal neoplasia (CRN) in patients on antithrombotic agents.

Postprocedural bleeding is one of the most common severe complications of ER. We previously reported that anticoagulant use increased the risk of bleeding after colorectal endoscopic submucosal dissection (ESD) [3]. Many previous studies have also reported that the use of antithrombotic agents is a risk factor for bleeding after ER for gastrointestinal neoplasia [4–8]. Management of antithrombotic agents in patients undergoing ER has become an important issue; thus, guidelines for antithrombotic agent management in the perioperative period of ER have been developed [9–12]. The American Society for Gastrointestinal Endoscopy [9], the European Society of Gastrointestinal Endoscopy, the British Society for Gastroenterology [10], and the previous Japan Gastroenterological Endoscopy Society (JGES) guidelines [11] recommended that warfarin should be temporarily discontinued and bridge therapy with heparin should be performed during gastroenterological endoscopic procedures with a high risk of bleeding in patients on warfarin therapy.

Recently, several studies have indicated a significantly increased risk of bleeding in patients who underwent heparin replacement instead of continued warfarin therapy in the perioperative period of ER [5, 13–16], and there has been some discussion as to whether warfarin should be replaced with heparin in the perioperative period of ER. The JGES guidelines [12] recommended that continued warfarin treatment should be considered during gastroenterological endoscopic procedures with a high risk of bleeding as an alternative to heparin replacement in patients on warfarin therapy. However, there is insufficient evidence to support the management of warfarin for colorectal ER, and further studies are needed. Therefore, we performed the present study to verify the risk of bleeding after ER for CRNs in patients with continued warfarin use.

## 2. Patients and Methods

### 2.1. Patients

**Figure 1:**
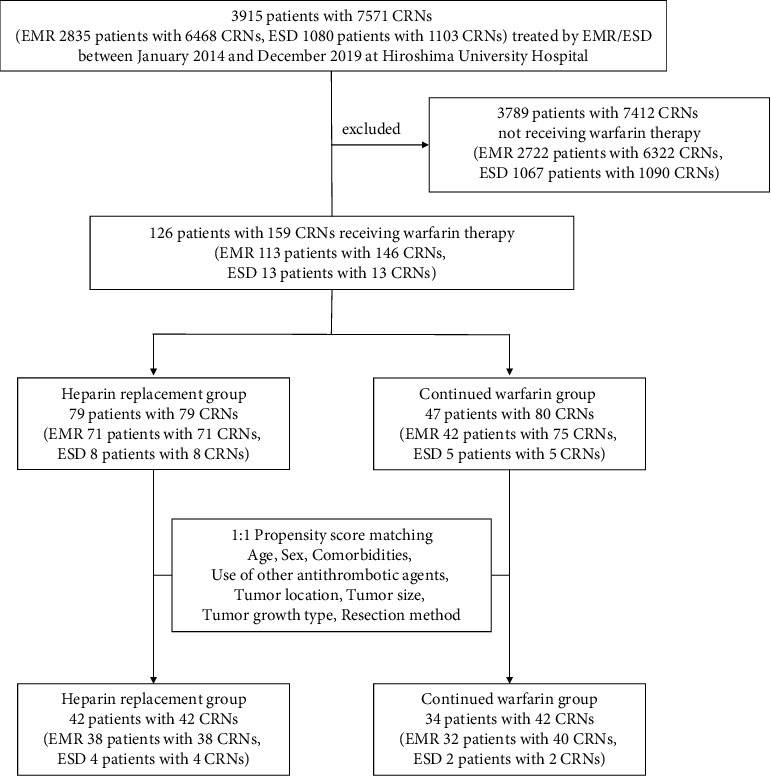
Patient enrollment in the present study. CRNs: colorectal neoplasia; EMR: endoscopic mucosal resection; ESD: endoscopic submucosal dissection.

We performed endoscopic mucosal resection (EMR) or ESD in 3915 patients with 7571 CRNs at Hiroshima University Hospital between January 2014 and December 2019. Among the patients, 3789 patients with 7412 CRNs who did not receive warfarin therapy were excluded (shown in Figure 1). The remaining 126 patients with 159 CRNs who received warfarin therapy were included in the present study and assessed retrospectively. EMR was performed in 113 patients with 146 CRNs, whereas ESD was performed in 13 patients with 13 CRNs. Patients were divided into two groups: 79 patients with 79 CRNs with heparin replacement (heparin replacement group) and 47 patients with 80 CRNs with continued warfarin use (continued warfarin group). All clinical data were retrospectively obtained from medical records. The following information was obtained for all patients: age, sex, laboratory test results, including hemoglobin level, comorbidities, use of anticoagulants and antiplatelet agents, information regarding cessation of antithrombotic agents, treatment with heparin replacement, details of endoscopic findings and procedures, and pathological findings of the resected specimens.

The present study was performed in accordance with the ethical standards laid down in the Declaration of Helsinki and its later amendments. This study protocol was reviewed and approved by the Institutional Review Board of Hiroshima University Hospital (approval number E-1064, registration date: January 16, 2017). Written informed consent was obtained from all patients.

### 2.2. Management of Warfarin

Before April 2017, we performed ER with heparin replacement according to the previous JGES guidelines [11] in all cases. Warfarin was replaced with heparin 3–5 days before the procedure. The heparin dose was adjusted to attain the required activated partial thromboplastin time (APTT). According to the guideline, heparin was started intravenously at a dose of 10,000-20,000 U/day, and the heparin dose was adjusted to attain the target range of APTT (1.5-2.5 times the reference control value) [11]. Heparin was suspended for at least 3 h before the procedure. The day after the procedure, once hemostasis had been confirmed, warfarin was restarted at the prewithdrawal dose. Heparin was discontinued when the prothrombin time-international normalized ratio (PT-INR) returned to the therapeutic range. After April 2017, we performed ER with continued warfarin use without heparin replacement according to the latest JGES guidelines [12] in all cases. We measured the PT-INR on the morning of the procedure to confirm that the PT-INR was within the therapeutic range and thereafter performed ER with the continued warfarin use. According to the guideline, the target ranges of PT-INR in patients aged ≥70 years with nonvalvular atrial fibrillation and in other patients were 1.6-2.6 and 2.0-3.0, respectively [17].

### 2.3. Indications and EMR Procedure

Indications of EMR for CRNs followed the JGES guidelines for colorectal ESD/EMR [18]. ER is recommended for CRNs ≥ 6 mm in size and superficial depressed-type CRNs ≤ 5 mm in size.

EMR was performed by multiple endoscopists with more than 1000 conventional endoscopic experiences using a high-resolution video colonoscope (CF-H260AZI, PCF-Q260AZI, CF-HQ290ZI, or PCF-H290ZI (Olympus, Tokyo, Japan)) or a gastroscope (GIF-Q260J (Olympus)) for rectal lesions. We used a 10% glycerin solution containing a small amount of indigo carmine (indigo carmine/glycerol: 2 mL/200 mL) as the injection solution. A spiral snare, SnareMaster (Olympus), or Captivator II Snare (Boston Scientific, Boston, MA, USA) was used; selection was dependent on the tumor size or the particular situation. At the end of the procedure, hemostatic clips were rarely used, and hemostatic forceps were used when post-EMR hemostasis was necessary. All exposed vessels on the resected ulcer were coagulated using hemostatic forceps (Coagrasper (Olympus) or HDB2418W-W (Pentax, Tokyo, Japan)) in conjunction with a high-frequency generator (ESG-100 or ESG-300 (Olympus)). We performed a blood examination in all patients the day after EMR. If the blood examination and abdominal findings were within normal range and regular, the patient was permitted to eat a meal and leave the hospital the same day.

### 2.4. Indications and ESD Procedure

Indications of ESD for CRNs followed the JGES guidelines for colorectal ESD/EMR [18]. ESD is indicated for lesions, such as laterally spreading tumors of the nongranular type, particularly, the pseudodepressed type, tumors with a V_I_-type pit pattern, carcinomas with shallow submucosal invasion, large depressed-type tumors, large protruded-type tumors suspected to be carcinoma, and mucosal tumors with submucosal fibrosis.

ESD was performed by five expert endoscopists using a high-resolution video colonoscope (CF-H260AZI, PCF-Q260AZI, CF-HQ290ZI, PCF-H290ZI, or PCF-H290TI (Olympus)) or a gastroscope (GIF-Q260J, Olympus) for rectal lesions. We used a 10% glycerin solution containing 0.4% sodium hyaluronate (MucoUp; Johnson & Johnson, New Brunswick, NJ, USA) and a small amount of indigo carmine (indigo carmine/hyaluronate/glycerol: 0.2 mL/10 mL/10 mL) as the injection solution. We used DualKnife (Olympus) or DualKnife J (Olympus). An ITknife nano (Olympus) or SB Knife Jr (Sumitomo Bakelite, Tokyo, Japan) was used depending on the situation. At the end of the procedure, hemostatic clips were rarely used, and hemostatic forceps were used when post-ESD hemostasis was necessary. At the end of the procedure, all exposed vessels on the resected ulcer were coagulated using the same devices used in EMR. Blood examination was performed in all patients the day after ESD. If the blood examination and abdominal findings were within normal range and regular, the patient was permitted to eat a light meal and left the hospital within a few days.

### 2.5. Evaluation

Clinicopathological characteristics (age, sex, comorbidities, use of other antithrombotic agents, tumor location, tumor size, tumor growth type, resection method, and pathological diagnosis) and bleeding rate after ER were assessed. The clinicopathological characteristics and bleeding rate after ER were compared between the heparin replacement group and continued warfarin group with propensity score matching analysis. In addition, the characteristics of patients with bleeding after ER were examined.

Bleeding after ER was defined as apparent bleeding, massive melena, or a ≥2 g/dL decrease in hemoglobin level compared with the last preoperative level according to a previous report [19]. Colonoscopy was performed when bleeding after ER was suspected. Hemostatic forceps, rather than endoclips, were used, and hemostasis was applied if bleeding points could be identified. A blood transfusion was administered if excessive bleeding with hemorrhagic shock and/or markedly decreased blood hemoglobin levels (<8 g/dL) were observed.

In all cases of bleeding after ER, findings such as active bleeding, coagulation adhesion, and exposed vessels were observed, and the lesion causing the bleeding could be identified. Therefore, the bleeding event was counted per polyp in this study.

### 2.6. Statistical Analysis

Quantitative data are shown as the mean ± standard deviation or percentage. Differences in categorical variables were analyzed using Fisher's exact test or chi-squared test. Differences in continuous variables were analyzed using Student's *t*-test or Mann–Whitney *U* test. Statistical significance was set at *P* < 0.05.

Propensity score matching analysis was performed to reduce the influence of possible confounding factors. To estimate the propensity score, we fitted a logistic regression model, and the following variables were included in the model as covariates: age, sex, comorbidities, use of other antithrombotic agents, tumor location, tumor size, tumor growth type, and resection method. The variables were carefully selected based on previous studies [3, 4, 7, 8, 20–24]. We calculated the *C*-statistic to evaluate the goodness of fit. After the propensity scores were estimated, one-to-one nearest-neighbor matching was performed based on a matching algorithm with a 0.2 caliper width. We used standardized difference to measure covariate balance. JMP statistical software (version 15.0.0; SAS Institute, Cary, NC) was used for all statistical analyses.

## 3. Results

### 3.1. Clinicopathological Characteristics of Patients

Table 1 shows the clinical characteristics of the enrolled patients on warfarin therapy and comparison of the characteristics between the two groups. Before propensity score matching, the mean age was significantly higher in the heparin replacement group (72.3 ± 8.0 years) than in the continued warfarin group (69.0 ± 8.5 years) (*P* = 0.0254). The proportion of patients with comorbidities was 87.3% (69/79) in the heparin replacement group and 91.5% (43/47) in the continued warfarin group. The proportion of patients with congestive heart failure was significantly higher in the heparin replacement group (11.4%, 9/79) than in the continued warfarin group (0%, 0/47) (*P* = 0.0259). There was no significant difference between the two groups in the proportion of patients with other comorbidities. In total, the proportion of patients receiving other antithrombotic agents was 28.6% (36/126) and the proportion of patients receiving aspirin was the highest among other antithrombotic agents (15.9%, 20/126). There was no significant difference between the two groups in the proportion of patients receiving other antithrombotic agents.  Table 2 shows the baseline characteristics of the enrolled CRNs and a comparison of the characteristics between the two groups. EMR was performed in 91.8% (146/159) of CRNs, whereas ESD was performed in 8.2% (13/159) of CRNs. Before propensity score matching, there was no significant difference between the two groups in tumor location, tumor growth type, resection method, and pathological diagnosis. The mean tumor size was significantly larger in the heparin replacement group (13.4 ± 16.3 mm) than in the continued warfarin group (8.0 ± 6.4 mm) (*P* = 0.0064).

The *C*-statistic for goodness of fit was 0.82002 in the propensity score model. After propensity score matching, only the rate of patients with chronic kidney disease was significantly higher in the continued warfarin group (64.7%, 22/34) than in the heparin replacement group (26.2%, 11/42) (*P* = 0.0011). There was no significant difference between the two groups in other clinicopathological characteristics (Table 2).

### 3.2. Bleeding Rate after ER

Table 3 shows the comparison of the bleeding rate after ER between the two groups. Before propensity score matching, the bleeding rate after ER was 5.7% (9/159) in total. The bleeding rate after ER was significantly higher in the heparin replacement group (10.1%, 8/79) than in the continued warfarin group (1.3%, 1/80) (*P* = 0.0178). In the continued warfarin group, there was only one patient who had bleeding after ER. The bleeding rate after ER was significantly higher in ESD (23.1%, 3/13) than in EMR (4.1%, 6/146) (*P* = 0.0272). After propensity score matching, the bleeding rate after ER was significantly higher in the heparin replacement group (11.9%, 5/42) than in the continued warfarin group (0%, 0/42) (*P* = 0.0211).

### 3.3. Characteristics of Cases with Bleeding after ER

The characteristics of patients with bleeding after ER are shown in Table 4. In the heparin replacement group, bleeding after ER occurred in eight patients; most were male (87.5%, 7/8), and most of the CRNs were the protruded type (87.5%, 7/8). More than half of the patients (75%, 6/8) had multiple comorbidities, and half (50%, 4/8) used other antithrombotic agents. EMR was performed in five patients, and ESD was performed in three patients. In two patients who underwent ESD, CRNs were large lesions with a tumor size of 100 mm. Bleeding occurred within 2 days after ER in most patients (87.5%, 7/8). One patient bled nine times; this patient used two antithrombotic agents, and the CRN was a 100 mm sized T1 carcinoma located in the rectum. In the continued warfarin group, bleeding after ER occurred in only one patient. The patient did not use any other antithrombotic agents, and the CRN was a 6 mm sized adenoma located in the rectum. In all patients, colonoscopy was performed after bleeding, all exposed bleeding vessels on the artificial ulcer were coagulated, and hemostasis was applied. No patients required blood transfusion or experienced ischemic events perioperatively.

## 4. Discussion

Our data showed that the risk of bleeding after colorectal ER was significantly lower in patients with continued warfarin use than in those with heparin replacement. We previously reported that the risk of bleeding after gastric ESD was higher in patients who took warfarin and received heparin bridging and those who took direct oral anticoagulants (DOACs) than in those who did not [25]. Moreover, we previously reported that the risk of bleeding after colorectal ESD was higher in patients who took anticoagulants than in those who did not [3]. In the present study, we compared the bleeding rate after ER for CRNs in patients with continued warfarin use and in patients with heparin replacement instead of warfarin with a propensity score matching analysis. To our knowledge, this is the first study to compare the risk of bleeding after colorectal ER between patients with continued warfarin use and those with heparin replacement. The results of our study are consistent with and supportive of the latest JGES guidelines [12].

Previous studies have indicated that heparin replacement increases the risk of postprocedural bleeding in patients undergoing invasive procedures, such as colorectal and gastric ER, surgical procedures, and cardiac device implantation [5, 13–16, 26–30]. Some studies have reported a comparison of postprocedure bleeding risk between patients with continued warfarin use and those with heparin replacement [16, 30]. Biase et al. [30] reported that compared to heparin replacement, continued warfarin treatment resulted in a significantly lower incidence of bleeding after catheter ablation of atrial fibrillation in a randomized trial. Harada et al. [16] reported that the bleeding rate after gastric ESD was lower in patients with continued warfarin use than in patients with heparin replacement in a single-center retrospective analysis. The reasons why heparin replacement was associated with higher postprocedural bleeding risk have been discussed in several previous studies. Du et al. [26] reported that overlap of oral anticoagulants and heparin may increase the risk of bleeding. Robinson et al. [31] reported that the reason may be explained by the concept of an “anticoagulant stress test.” In other words, with warfarin, intraoperative hemostasis may occur at the site of potential bleeding, leading to decreased bleeding after the procedure. Hirsh et al. [32] reported that according to the mechanism of action, heparin in combination with antithrombin III is known to exert not only anticoagulant but also antiplatelet actions via the antithrombin effect, and heparin products could bind to platelets, inhibit platelet function, and contribute to hemorrhagic effects.

In the present study, no patients experienced thromboembolic events during the perioperative period. A patient with a mechanical aortic valve who does not receive anticoagulation medication has an annual stroke risk of approximately 4% [33]. Blacker et al. [34] reported that stroke occurred in 1.06% (12/1137) of endoscopic procedures with regard to thromboembolic complications associated with warfarin withdrawal. Therefore, interruption of anticoagulation in the perioperative period, even for a short period, may be unacceptable in patients with moderate-to-high risk of thromboembolic events. Heparin has been used as a substitute for anticoagulants. Several studies have shown that heparin replacement does not reduce the risk of thromboembolic events compared with continued oral anticoagulants in procedures, such as cardiac device implantation and surgery [26, 28, 35]. Moreover, Nagata et al. [15] reported that the risk of thromboembolism after high-risk endoscopic procedures was higher in patients who received warfarin plus heparin replacement or DOACs plus heparin replacement than in those with DOACs alone. Therefore, heparin replacement may not be superior to continued warfarin use in terms of bleeding and thromboembolic events.

The latest JGES guidelines [12] suggest a temporary shift from warfarin to DOACs in patients with nonvalvular atrial fibrillation during gastroenterological endoscopic procedures with a high risk of bleeding. We previously reported that bleeding after colorectal ESD occurred in 26.3% (5/19) and 22.0% (2/9) of patients taking warfarin and DOAC, respectively [3]. Nagata et al. [15] reported that the rate of bleeding after high-risk endoscopic procedures was significantly higher in warfarin users than in DOAC users. Conversely, Harada et al. [36] reported that the bleeding rate after colorectal ESD was higher in DOAC users (16.0%, 4/25) than in warfarin users (7.7%, 2/26). Whether warfarin or DOAC has a higher risk of bleeding is still controversial, and thus, further studies are needed.

Some studies have discussed various risk factors for bleeding after ER, in addition to anticoagulant and heparin replacement [3, 4, 7, 8, 20–24, 37]. We previously reported that continued use of low-dose aspirin increased the risk of bleeding after colorectal ESD compared with the nonuse of antithrombotic agents [37]. Several studies have reported that taking antiplatelet agents is a risk factor for bleeding after colorectal ER [4, 8]. In addition, we previously reported that four out of five patients on warfarin therapy who bled after colorectal ESD took warfarin as well as antiplatelet agents [3]. Takeuchi et al. [7] also reported that combination therapy with low-dose aspirin and warfarin was a significant risk factor for bleeding after gastric ESD. In the present study, four of the nine patients who bled after ER took warfarin as well as antiplatelet agents. It has been suggested that patients with antiplatelet agents in addition to anticoagulants are at a high risk of bleeding. We also reported that the location of lesions in the rectum was a significant independent risk factor for delayed bleeding after colorectal ESD [20]. In our study, two of the nine CRNs that bled after ER were located in the rectum. Some studies have reported that large tumor size is a risk factor for bleeding after colorectal ER [4, 8, 21–24]. In the present study, all three CRNs that bled after ESD were large-sized lesions. Niikura et al. [4] reported that colorectal ESD was a significant risk factor for bleeding after colorectal ER. In our study, ESD had a higher post-ER bleeding rate than EMR, consistent with a previous study finding. The patient who had nine bleeding episodes was taking two antiplatelet agents in addition to warfarin, and the CRN was a 100 mm lesion located in the rectum; thus, the patient had multiple bleeding risk factors. Bleeding risk factors including not only use of antithrombotic agents but also other factors should be comprehensively considered, and ER and postoperative management should be conducted with sufficient attention.

There are no standardized endoscopic methods for preventing bleeding after ER. In our hospital, all exposed vessels on the resected ulcer were coagulated with hemostatic forceps in conjunction with a high-frequency generator after colorectal ER. Lee et al. [38] reported that prophylactic cautery of visible vessels reduced postcolorectal EMR bleeding. Furthermore, several previous studies reported that closure of mucosal defects with clips after EMR for large colorectal lesions decreased the risk of bleeding after ER [39, 40]. Several techniques, such as endoloop [41], hand-suturing [42], OverStitch endoscopic suturing [43], 8-ring [44], mucosal incision [45], over-the-scope clip [46], and two-channel techniques [47], for complete closure of large, resected ulcers after ER have been demonstrated. The tissue shielding method using a polyglycolic acid (PGA) sheet and fibrin glue may effectively prevent bleeding. Two prospective nonrandomized studies have shown favorable results for the PGA sheet shielding method in gastric ER [48, 49].

Our study has some limitations. First, this was a single-center, retrospective cohort study based on clinical records. We used propensity score matching analysis to minimize selection bias; however, we could not eliminate bias. Second, the sample size was relatively small. Our study only enrolled patients who underwent colorectal EMR/ESD; patients who underwent other ERs, such as polypectomy and underwater EMR, were excluded. A multicenter prospective study, with a larger number of cases, should be conducted in future to confirm the latest JGES guidelines. Third, the “heparin replacement” strategy was implemented in earlier years (2014-2017) and the “continued warfarin” strategy in later years (2017-2019). The timing of treatment was different between the two groups, and bias resulting from advances in endoscopic scopes and proficiency of endoscopists may have occurred.

## 5. Conclusions

The risk of bleeding after colorectal ER was significantly lower in patients with continued warfarin use than in those with heparin replacement. Our data supports the recommendations of the latest JGES guidelines for patients receiving warfarin therapy.

## Figures and Tables

**Table 1:** Clinical characteristics of enrolled patients on warfarin therapy.

Variables	Total (*n* = 126)	Heparin replacement (*n* = 79)	Continued warfarin (*n* = 47)	*P* value
Age, mean ± SD, years old	71.1 ± 8.2	72.3 ± 8.0	69.0 ± 8.5	0.03
Sex, male	91 (72.2)	53 (67.1)	38 (80.9)	0.10
Comorbidities				
Yes	112 (88.9)	69 (87.3)	43 (91.5)	0.57
Atrial fibrillation	49 (38.9)	35 (44.3)	14 (29.8)	0.13
Ischemic heart disease	24 (19.0)	14 (17.7)	10 (21.3)	0.64
Congestive heart failure	9 (7.1)	9 (11.4)	0 (0)	0.03
Cerebral infarction	20 (15.9)	13 (16.5)	7 (14.9)	1
Hypertension	52 (41.3)	31 (39.2)	21 (44.7)	0.58
Diabetes mellitus	39 (31.0)	24 (30.4)	15 (31.9)	1
Chronic kidney disease	63 (50.0)	34 (43.0)	29 (61.7)	0.06
Chronic liver disease	15 (11.9)	10 (12.7)	5 (10.6)	1
Blood disorder	2 (1.6)	2 (2.5)	0 (0)	0.53
Use of other antithrombotic agents				
Yes	36 (28.6)	25 (31.6)	11 (23.4)	0.42
Aspirin	20 (15.9)	13 (16.5)	7 (14.9)	1
Clopidogrel	7 (5.6)	5 (6.3)	2 (4.3)	1
Others	13 (10.3)	9 (11.4)	4 (8.5)	0.77

SD: standard deviation.

**Table 2:** Characteristics of enrolled CRNs.

Variables	Before propensity score matching				*P* value	After propensity score matching				*P* value
	Total (*n* = 159)	Heparin replacement (*n* = 79)	Continued warfarin (*n* = 80)	Standardized difference		Total (*n* = 84)	Heparin replacement (*n* = 42)	Continued warfarin (*n* = 42)	Standardized difference	
Tumor location										
Colon	143 (89.9)	73 (92.4)	70 (87.5)	0.16	0.43	81 (96.4)	40 (95.2)	41 (97.6)	0.13	1
Rectum	16 (10.1)	6 (7.6)	10 (12.5)			3 (3.6)	2 (4.8)	1 (2.4)		
Tumor size, mean ± SD (mm)	10.6 ± 12.6	13.4 ± 16.3	8.0 ± 6.4	0.43	0.006	9.3 ± 7.9	9.8 ± 8.6	8.7 ± 6.9	0.15	0.50
Tumor growth type										
Protruded	116 (73.0)	57 (72.2)	59 (73.8)	0.04	0.86	61 (72.6)	29 (69.0)	32 (76.2)	0.16	0.63
Superficial	43 (27.0)	22 (27.8)	21 (26.2)			23 (27.4)	13 (31.0)	10 (23.8)		
Resection method										
EMR	146 (91.8)	71 (89.9)	75 (93.8)	0.14	0.37	78 (92.9)	38 (90.5)	40 (95.2)	0.18	0.68
ESD	13 (8.2)	8 (10.1)	5 (6.2)			6 (7.1)	4 (9.5)	2 (4.8)		
Pathological diagnosis										
Adenoma	145 (91.2)	71 (89.9)	74 (92.5)	0.09		79 (94.0)	39 (92.8)	40 (95.2)	0.10	
Tis carcinoma	11 (6.9)	6 (7.6)	5 (6.3)	0.05	0.78	3 (3.6)	2 (4.8)	1 (2.4)	0.13	0.84
T1 carcinoma	3 (1.9)	2 (2.5)	1 (1.2)	0.09		2 (2.4)	1 (2.4)	1 (2.4)	0	

SD: standard deviation; CRN: colorectal neoplasia; EMR: endoscopic mucosal resection; ESD: endoscopic submucosal dissection.

**Table 3:** Bleeding rate after ER before and after propensity score matching.

Resection method	Before propensity score matching			After propensity score matching		
	Total (*n* = 159)	Heparin replacement (*n* = 79)	Continued warfarin (*n* = 80)	Total (*n* = 84)	Heparin replacement (*n* = 42)	Continued warfarin (*n* = 42)
EMR	6/146 (4.1)^a^	5/71 (7.0)	1/75 (1.3)	5/78 (6.4)	5/38 (13.1)	0/40 (0)
ESD	3/13 (23.1)^b^	3/8 (37.5)	0/5 (0)	0/6 (0)	0/4 (0)	0/2 (0)
Total	9/159 (5.7)	8/79 (10.1)^c^	1/80 (1.3)^d^	5/84 (6.0)	5/42 (11.9)^e^	0/42 (0)^f^

EMR: endoscopic mucosal resection; ESD: endoscopic submucosal dissection; ER: endoscopic resection. a vs. b: *P* < 0.05, c vs. d: *P* < 0.05, and e vs. f: *P* < 0.05.

**Table 4:** Characteristics of cases with bleeding after procedure.

No. of cases	Age (years)	Sex	Comorbidities	Use of other antiplatelets	Type of procedure	Tumor location	Tumor size (mm)	Tumor growth type	Pathological diagnosis	Bleeding date after procedure (day)	Bleeding frequency
Heparin replacement group											
1	65	Male	Atrial fibrillation, spinal infarction	Aspirin	EMR	A	5	Protruded	Adenoma	2	4
2	63	Male	Atrial fibrillation, chronic hepatitis B	Aspirin	EMR	S	6	Protruded	Adenoma	1	1
3	79	Male	Atrial fibrillation	None	EMR	A	8	Superficial	Adenoma	1	1
4	68	Male	Atrial fibrillation	None	EMR	S	8	Protruded	Adenoma	2	2
5	48	Male	Cardiac hypertrophy, cirrhosis B	None	EMR	S	6	Protruded	Adenoma	2	1
6	73	Male	Myocardial infarction, ventricular aneurysm	Aspirin, prasugrel	ESD	R	100	Protruded	T1 carcinoma	1, 8, 11, 12, 16, 17, 19, 21, 23	9
7	81	Male	Atrial fibrillation, myocardial infarction, cerebral infarction, chronic kidney disease (hemodialysis)	None	ESD	A	100	Protruded	Tis carcinoma	8	1
8	70	Female	Atrial fibrillation, cerebral infarction, chronic kidney disease	Ticlopidine	ESD	T	40	Protruded	Adenoma	2	2
Continued warfarin group											
1	64	Male	Deep vein thrombosis	None	EMR	R	6	Protruded	Adenoma	2	1

A: ascending colon; EMR: endoscopic mucosal resection; ESD: endoscopic submucosal dissection; R: rectum; S: sigmoid colon; T: transverse colon.

## Data Availability

All data used to support the findings of this study are included in this article.
